# Fine root dynamics in lodgepole pine and white spruce stands along productivity gradients in reclaimed oil sands sites

**DOI:** 10.1002/ece3.1742

**Published:** 2015-10-02

**Authors:** Ghulam Murtaza Jamro, Scott X. Chang, M. Anne Naeth, Min Duan, Jason House

**Affiliations:** ^1^ Department of Renewable ResourcesUniversity of AlbertaEdmontonAlbertaT6G 2E3Canada; ^2^ Department of Soil ScienceSindh Agriculture UniversityTandojamSindh70060Pakistan

**Keywords:** Boreal forest, fine root, ingrowth core, oil sands reclamation, overburden, sequential coring, tailings sand

## Abstract

Open‐pit mining activities in the oil sands region of Alberta, Canada, create disturbed lands that, by law, must be reclaimed to a land capability equivalent to that existed before the disturbance. Re‐establishment of forest cover will be affected by the production and turnover rate of fine roots. However, the relationship between fine root dynamics and tree growth has not been studied in reclaimed oil sands sites. Fine root properties (root length density, mean surface area, total root biomass, and rates of root production, turnover, and decomposition) were assessed from May to October 2011 and 2012 using sequential coring and ingrowth core methods in lodgepole pine (*Pinus contorta* Dougl.) and white spruce (*Picea glauca* (Moench.) Voss) stands. The pine and spruce stands were planted on peat mineral soil mix placed over tailings sand and overburden substrates, respectively, in reclaimed oil sands sites in Alberta. We selected stands that form a productivity gradient (low, medium, and high productivities) of each tree species based on differences in tree height and diameter at breast height (DBH) increments. In lodgepole pine stands, fine root length density and fine root production, and turnover rates were in the order of high > medium > low productivity sites and were positively correlated with tree height and DBH and negatively correlated with soil salinity (*P *<* *0.05). In white spruce stands, fine root surface area was the only parameter that increased along the productivity gradient and was negatively correlated with soil compaction. In conclusion, fine root dynamics along the stand productivity gradients were closely linked to stand productivity and were affected by limiting soil properties related to the specific substrate used for reconstructing the reclaimed soil. Understanding the impact of soil properties on fine root dynamics and overall stand productivity will help improve land reclamation outcomes.

## Introduction

Fine roots as part of the tree root system play an important role in resource (e.g., water and nutrients) capture (West et al. 2004). It is widely accepted that fine root production, turnover, and decomposition make a greater contribution to available soil nutrient pools than inputs from aboveground litter to the soil (Aerts et al. 1992). Fine root length density and root surface area are key root morphological features and have an important role to play in soil resource exploitation (Gilroy and Jones 2000; Metcalfe et al. 2008). Fine root length density can be used to estimate the ability of roots to proliferate and to sequester nutrients, whereas root surface area can be used to estimate the stand absorptive potential for resources (Eissenstat et al. 2000). Thus, enhanced fine root growth can assist in nutrient retention and scavenging and mining resource for acquisition from the soil (Hinsinger et al. 2005; Lambers et al. 2008), and it would be particularly important in reclaimed ecosystems where availabilities of resources such as water and nutrients are often limiting (Jung et al. 2014; Boldt‐Burisch et al. 2015; Duan et al. 2015). Alterations in fine root growth and architectural traits may reflect the availability of soil resources (Rosenvald et al. 2011) and stand characteristics (Jung and Chang 2013). Thus, the proliferation of fine roots within a stand may serve as a useful indicator for assessing stand productivity in reclaimed ecosystems (Gilroy and Jones 2000).

Surface‐mining activities in the Athabasca oil sands region (AOSR) have disturbed about 750 km^2^ of land, which accounts for about 0.2% of the mixed wood boreal forest ecosystem (Government of Alberta 2014). Oil sands companies are legally bound to reclaim the disturbed land according to the Alberta Environmental Protection and Enhancement Act (Powter et al. 2012). Current oil sands reclamation practices predominantly involve the use of peat mineral soil mix (PMM) as an organic capping material over tailings sand or overburden substrates (Rowland et al. 2009). Inherent properties of these materials, such as the slow decomposition rate of PMM, means that it releases nutrients (e.g., nitrogen) slowly due to a wide carbon to nitrogen ratio (Jamro et al. 2014; Kwak et al. 2015). The substrates below the capping materials may have high soluble salt concentrations, poor drainage, and heavy compaction, which could limit the growth of trees (Jung et al. 2014; Duan et al. 2015) and fine roots (Strand et al. 2008).

Processes associated with fine root dynamics such as fine root production and turnover are thought to be some of the main drivers of biogeochemical nutrient cycling and overall stand productivity in terrestrial ecosystems (Yuan and Chen 2013; Gundale et al. 2014; Tripathi et al. 2014). These processes are responsive to soil environmental changes (Jagodziński and Kałucka 2010; Yuan and Chen 2013) and reclamation practices (Lazorko and Van Rees 2012; Jung et al. 2014). However, how reclamation practices influence fine root dynamics and their relationship with stand productivity is poorly understood in oil sands reclamation. In a recent study, Jung et al. (2014) reported that tailings sand and overburden substrates differ in pore size distribution, with tailings sand having more macropores with low water‐ and nutrient‐holding capacity and the overburden material having more micropores. These differences in pore structure in the soil may influence the distribution of resources through enhanced leaching of nutrients in tailings sand and decreased drainage in overburden material that may induce an anaerobic environment and limit nutrient transformation rates and their availability (Brady and Weil 2008), affecting fine root growth. The overburden material can be severely compacted, which could lead to increased bulk density and decreased root growth (Jung et al. 2014). Overburden material can be saline sodic, containing sodium, sulfate, and chloride ions (Lazorko and Van Rees 2012), which can cause imbalances in water and nutrient availabilities (Munns and Tester 2008). Soil salinity could alter the morphology of fine roots (Lazorko and Van Rees 2012) and reduce fine root biomass (Jung et al. 2014). Therefore, the life span (Self et al. 1995), turnover rate and decomposition of fine roots may be affected (Zhang et al. 2009). The inherent soil properties of these substrates such as high salinity, compaction, nutrient, and water limitations are of concern regarding the sustainability of current oil sands reclamation practices because these soil factors affect the productivity of trees grown on reclaimed soils (Lilles et al. 2012; Duan et al. 2015; Pinno and Hawkes 2015).

In a recent study, Duan et al. (2015) found that the differences in stand productivity in oil sands reclamation were associated with differences in soil electrical conductivity (EC) and bulk density in overburden sites and volumetric water content in tailings sand sites. Electrical conductivity and bulk density were greater in low than in medium and high productivity sites planted to white spruce (*Picea glauca* (Moench.) Voss) on an overburden substrate, while volumetric water content was greater in high and medium than in low productivity sites planted to lodgepole pine (*Pinus contorta* Dougl) on a tailings sand substrate. Thus, fine root growth and their dynamics would likely be greater in high than in medium and low productivity sites. Although a few studies on fine root distribution of boreal forest species in oil sands reclamation have been conducted (Lazorko and Van Rees 2012; Jung et al. 2014), no one has assessed whether fine root dynamics might be related to stand productivity in oil sands reclamation. Understanding fine root dynamics is important for improving current reclamation practices for establishing functional forest ecosystems in the oil sands (Yan et al. 2012; Jung and Chang 2013; Jung et al. 2014).

The objective of this study was to evaluate the relationship between fine root dynamics, including processes such as biomass production, turnover, decomposition, and morphological characteristics such as fine root surface area, root length density, and stand productivity in reclaimed oil sands soils. We hypothesized that fine root growth would have a positive relationship with aboveground tree growth along productivity gradients of different tree species planted in reclaimed oil sands sites. It was assumed that the differences in fine root dynamics would reflect the differences in inherited characteristics of tailings sand and overburden materials used for oil sands reclamation.

## Materials and Methods

### Site description and research plots

This study was conducted on an oil sands lease area reclaimed after open‐pit mining, located approximately 24 km north of Fort McMurray in the AOSR. The area has a continental boreal climate where winters are long and cold and summers are short and warm. The long‐term mean annual temperature was 1.0°C from 1981 to 2010 with a daily average temperature from −17.4°C in January to 17.1°C in July. Mean annual precipitation was 418.6 mm, which mostly falls as rain (316.3 mm) during summer (Environment Canada 2015). The mean temperature was 16.7°C and 17.3 °C in 2011 and 2012 during the study. The total precipitation was 87 mm in 2011 and 280 mm in 2012 (data not shown), indicating a dry year in 2011.

The study sites were reclaimed with PMM as the organic capping material over tailings sand or overburden substrates. These sites were reclaimed at different times between 1984 and 1996. The selected sites had PMM depth ranging from 11 to 48 cm (Table 1). Lodgepole pine was planted on PMM over a tailings sand substrate and white spruce was planted on PMM over overburden substrate. The main understory plant species on lodgepole pine sites were prickly rose (*Rosa acicularis* Lindl), raspberry (*Rubus idaeus* L.), sweet clover (*Melilotus* spp.), dandelion (*Taraxacum officinale* L.), and slender wheat grass (*Agropyron trachycaulum* Link Malte). The understory vegetation in white spruce stands was dominated by willow (*Salix* spp.), green alder (*Alnus crispa* (Ait.) Pursh), sweet clover, dandelion, and bluejoint grass (*Calamagrostis canadensis* (Michx) Beau.) (Jung et al. 2014). Some key site characteristics are summarized in Table 1.

**Table 1:** Characteristics of studied reclaimed oil sands sites in the Athabasca oil sands region, Alberta, Canada

Site no.	Location		Year^a^	Substrate^b^	Tree species	Stand productivity	Amendment depth (cm)	Total C (g kg^−1^)	Soil compaction (kpa)^c^	Stand density (stem ha^−1^)
	Latitude	Longitude								
1	N 56°59′02″	W 111°27′04″	1996	Tailing sand	Lodgepole pine	Low	17	67	448	1500
2	N 56°58′38″	W 111°27″39″	1991	Tailing sand	Lodgepole pine	Low	14	65	620	2300
3	N 56°59′30″	W 111°27′15″	1996	Tailing sand	Lodgepole pine	Medium	14	50	517	1700
4	N 56°58′55″	W 111°29′58″	1992	Tailing sand	Lodgepole pine	Medium	30	79	483	2700
5	N 56°58′42″	W 111°27′51″	1991	Tailing sand	Lodgepole pine	High	18	62	276	2300
6	N 56°59′47″	W 111°28′14″	1991	Tailing sand	Lodgepole pine	High	24	160	255	2100
7	N 56°58′54″	W 111°31′04″	1992	Overburden	White spruce	Low	12	15	2137	2000
8	N 56°58′45″	W 111°27′24″	1991	Overburden	White spruce	Low	22	85	2137	2300
9	N 56°59′25″	W 111°27′04″	1996	Overburden	White spruce	Medium	30	51	1724	3100
10	N 56°59′09″	W 111°32′08″	1982	Overburden	White spruce	Medium	20	49	1792	2800
11	N 56°59′24″	W 111°32′09″	1991	Overburden	White spruce	High	11	45	1655	1900
12	N 56°59′51″	W 111°32′40″	1991	Overburden	White spruce	High	27	48	1517	2600

a Year indicates the year the trees were planted after soil reconstruction.

b Substrate below the organic capping material.

c Average of three soil layers (0–15, 15–30, and 30–45 cm).

A total of 12 sites were set up with six of each species (lodgepole pine and white spruce) encompassing three productivity levels (low, medium, and high). Initially, stands representing different productivity levels were identified based on visual inspection of tree performance and then the productivity of each stand was confirmed by tree height (using a height pole) and diameter at breast height (using a diameter tape) measurements that were used to calculate mean annual growth over time (Duan et al. 2015). Each productivity level was replicated twice for each species (Table 1) due to less availability of sites. For this study, 10 × 10 m plots were set up randomly at each site in June 2011. Tree age, stand density, and the degree of soil compaction were different at each site (Table 1). Tree age was 15–20 years on lodgepole pine sites and 15–29 years on white spruce sites. The stand density was 1500–2700 and 1900–3100 stems per hectare in pine and spruce sites, respectively. Soil compaction as measured by penetration resistance was 255–620 kpa in pine sites and 1517–2137 kpa in spruce sites (Table 1).

### Field and laboratory methods

Soil compaction was measured to a depth of 45 cm at three intervals (0–15, 15–30, and 30–45 cm) at five randomly selected locations in each plot using a soil penetrometer (Spectrum Technologies Inc., Aurora, IL) with a base tip of 1.27 cm diameter. Thermocouple temperature probes and CS616 time domain reflectometry (TDR) probes were installed at the depth of 10 cm below the soil surface in PMM and 10 cm below the PMM substrate interface, respectively, in each plot for the measurements of soil temperature and water content. These measurements were made hourly and datalogged.

Soil was sampled at 0–20 cm using a soil auger in five randomly selected locations in each plot and mixed to form a composite sample. The composited samples were stored in plastic bags and transported to the laboratory for analyses. Soil samples were sieved (2 mm), then homogenized manually after discarding the coarse fragments and roots, and used for analyses.

Soil gravimetric water content was measured after oven drying a subsample at 105°C for 24 h. Soil pH and EC were measured using a pH meter and an EC meter with a 1:2 (m:v) soil to water ratio (Kalra and Maynard 1991). Ammonium and nitrate concentrations were measured using the indophenol blue method (Keeney and Nelson 1982) and the vanadium oxidation method (Miranda et al. 2001), respectively, after extracting a subsample with 2 mol L^−1^ KCl solution (Mulvaney 1996). Total carbon and total nitrogen concentrations were analyzed by dry combustion with a Carlo Erba NA 1500 automated elemental analyzer (Carlo Erba Instruments, Millan, Italy).

### Fine root sampling and measurements

#### Fine root sampling

Both sequential and ingrowth core methods were used for root sampling (Vogt and Persson 1991). For the sequential soil core method, five soil samples were collected from randomly selected locations in each plot each month from June to September in 2011 and 2012. The sampling period was selected to measure the differences of fine root parameters during the growing season. The soil cores were collected at 0–30 cm in each plot using a steel corer (6.6 cm inner diameter). Samples were placed in plastic bags and transported to the laboratory in a cooler containing ice packs. For the ingrowth method, root ingrowth cores (30 cm long, 6.6 cm inner diameter) were constructed from plastic mesh (Quick Count plastic canvas, Uniek, Inc., Waunakee, WI) with an opening size of 1.5 × 1.5 mm. As most fine roots are located in the upper 30 cm (Yuan and Chen 2010), our root measurement was conducted for that depth. Twelve ingrowth cores were randomly installed to the 30 cm depth in each plot in July 2011. Before installing the cores, soil cores to 30 cm were taken using a steel corer. After removing roots from soils manually by sieving, the root‐free soils were placed back in the hole after an ingrowth core was inserted. Four ingrowth cores in each plot were retrieved in October 2011 and May and July 2012. Roots that penetrated through the ingrowth core and exposed were trimmed. The ingrowth core samples were placed in plastic bags and transported to the laboratory in a cooler containing ice packs.

#### Washing, sorting, and characterization of fine roots

Roots in sequential soil cores were separated from soils by washing them with tap water. Samples were soaked overnight, poured into trays, and rubbed gently. Roots floating on top of the water were collected by pouring water into a sieve (0.5 mm mesh) (Yuan and Chen 2013). The procedure was repeated until only rocks and organic debris (which floated more readily than root fragments) were left in soils. The roots in the sieve were poured in a plastic container and dispersed in water for further manual separation of roots from the organic material and washed again. Fine roots (<2 cm) were separated according to root vitality, that is, live and dead roots. Live roots were considered if they were pale in color on the surface and free of decay, while dead roots were brown or black in color and inflexible (Bennett et al. 2002; Brassard et al. 2011). Roots of pine and spruce trees were separated. Lodgepole pine roots were intact, reddish, and without signs of decomposition (Comeau and Kimmins 1989), while white spruce roots were generally orange colored without root hairs and longitudinal scar features or rills (Bohm 1979). The cleaned and fresh roots were scanned using a flatbed scanner set at 360 dpi. Root images were analyzed using WinRhizo image analysis software (Regent Instruments, Quebec, Canada) to determine the root length and surface area. Fine root length density was computed by dividing total root length per core by total volume of the core.

Each ingrowth core was processed separately after cutting into two pieces for ease of isolating the roots as roots were found in small amounts in each core. Isolation and assessment of roots followed the same procedure presented above.

#### Fine root biomass

After scanning, three root samples from sequential cores and two from ingrowth cores were oven‐dried at 70°C to constant weight and weighed. The sum of all root cores collected by sequential coring at each root sampling time in 2011 and 2012 was used for the measurement of total fine root biomass. Fine root biomass (kg ha^−1^) was calculated according to McClaugherty et al. (1982) as dry mass of living roots (gram) × 10^−3^ × 10^8^/ area of the core (area of the core: *πd* ^2^/4, *d* = 6.6 cm).

#### Fine root production

Fine root production was calculated for sequential and ingrowth core samples. Fine root production in ingrowth cores was estimated using fine root mass (sum of live and dead roots, as some roots died in the ingrowth cores in the incubation period and it was necessarily to combine them for true representation of fine root production) divided by the period of growth on a yearly basis (Vogt et al. 1998). In sequential cores, it was calculated by summing the root increments in sampling intervals in 2011 and 2012 (Fairley and Alexander 1985).

#### Fine root decomposition and turnover rates

Fine root decomposition rate was determined by the mesh bag technique (Mcclaugherty et al. 1984). A mesh bag, 10 × 20 cm in size, was made of fiber glass mesh with mesh size of 0.3 × 0.3 mm. Root samples collected from the top 30 cm of surface soil from the established plots of each site in June 2011 were used after being washed, dried at 65°C for 48 h, and cut into 2–5 cm lengths. Twelve mesh bags with 0.5 g of root materials were placed with ingrowth cores, at a depth of 20 cm with a slit in the soil cut to 45° to ensure good contact of the litter bag with the soil. Four bags from each plot were retrieved in October 2011 and May and July 2012. Residual root materials were carefully removed from the bags, washed in a sieve by pouring water slowly to remove adhered soil particles after brushing the mesh bag outside (if there were soil particles adhering to the litter bag), dried, and weighed. The weight loss upon drying was measured in each sample. Data of sampled mesh bags at each sampling time were pooled to represent a plot average. Decomposition rate constant (k) for each sample at each sampling time was calculated using the following exponential model based on the relationship between root mass remaining and incubation time of the mesh bag (Wieder and Lang 1982).

$$M_{t}/M_{0}=e^{-kt}$$

where *M*_0_ is the initial dry mass, *M*_*t*_ is the dry mass remaining at time *t*, and k is the decay constant and is expressed in years.

Mean residence time was calculated using the following equation (Giardina and Ryan 2000).

MRT=1/k

Turnover rates of each sample were calculated using the following formula (Yang et al. 2004):

Turnover rate (year^−1^) = annual fine root production (kg ha^−1^ year^−1^) / mean fine root biomass (kg ha^−1^).

### Statistical analyses

Repeated measures analysis of variance (ANOVA) was performed to determine whether time of sampling and stand productivity affected total fine root length density, mean root surface area, total fine root biomass, and root decomposition rate in each tree species using the PROC MIXED model. Sampling time was used as a repeated measures variable. One‐way ANOVA was performed to evaluate the effect of stand productivity level on fine root production and turnover rate separately for each method of root sampling. Only two replications of each productivity level were used due to limitations of site availability for each productivity gradient. Means were separated in both repeated measures and one‐way ANOVAs using Tukey's honestly significant difference test. Pearson correlation analysis was performed to examine the relationship between measured root parameters and soil properties (soil compaction, pH, EC, available nitrogen, volumetric water content, and soil temperature) for each species separately. Linear regression was conducted to determine the relationship between fine root dynamics (total fine root productivity, total turnover rate, and total fine root length density) and tree size (height and DBH) for each species. An *α* value of 0.05 was used to indicate significant differences in all analyses. Assumptions of normality and homogeneity of variance were tested with a Shapiro–Wilk test and Bartlett test when performing the ANOVA. All data were normally distributed except fine root production and turnover rate measured by the ingrowth core method, and data were log‐transformed to meet the assumption of normality and homogeneity. All statistical analyses were performed using version 9.2 of SAS (SAS Institute Inc., Cary, NC).

## Results

### Mean root surface area, total fine root length density, and total fine root biomass

Mean monthly root surface area (m^2^ m^−2^) in 2011 ranged from 0.53 (June) to 2.63 (August) and in 2012 ranged from 0.68 (June) to 3.04 (July), respectively, in lodgepole pine stands. Further, mean monthly root surface area across white spruce productivity gradient ranged from 0.52 (September) and 2.02 (June) in 2011 and from 0.65 (September) to 2.34 (July) in 2012, respectively, in white spruce stands, across the productivity gradient (Table 2). It was not affected by productivity level in pine stands in 2011 (*P = *0.27) and 2012 (*P *=* *0.29) although, in spruce stands, it was significantly influenced by productivity level and time of sampling (Tables 2, Table S1). Mean surface area was consistently greater in medium and high than in low productivity sites in both 2011 and 2012. Mean surface area decreased from June to September in both medium (−70%) and high (−33%) productivity levels but not in the low productivity level in both 2011 and 2012 based on data in Table 2.

**Table 2:** Fine root parameters of lodgepole pine and white spruce stands along stand productivity gradients in oil sands reclamation at different sampling times

Root parameter	Stand productivity	2011				2012			
		June	July	August	September	June	July	August	September
Root surface area (m^2^ m^−2^)									
Lodgepole pine	Low	0.53 (0.05)A^a^	0.77 (0.09)A	2.63 (0.53)A	1.66 (0.09)A	0.68 (0.02)A	1.32 (0.21)A	2.28 (0.92)A	1.86 (0.10)A
	Medium	1.77 (0.60)A	2.54 (0.46)A	1.81 (0.15)A	1.33 (0.11)B	1.92 (0.66)A	3.04 (0.81)A	1.86 (0.41)A	1.45 (0.15)AB
	High	2.06 (0.04)A	2.06 (0.42)A	1.72 (0.21)A	0.91 (0.10)C	2.31 (0.04)A	2.92 (0.05)A	1.72 (0.17)A	1.01 (0.17)B
White spruce	Low	0.97 (0.04)B	1.13 (0.06)A	0.73 (0.04)A	0.98 (0.08)A	1.13 (0.07)B	1.53 (0.06)B	0.93 (0.03)A	1.28 (0.22)A
	Medium	2.01 (1.30)A	1.92 (0.14)A	0.88 (0.06)A	0.52 (0.06)A	1.95 (0.05)A	2.34 (0.30)A	1.21 (0.15)A	0.65 (0.08)B
	High	1.38 (1.31)AB	1.67 (0.13)A	0.66 (0.03)A	0.85 (0.04)A	1.83 (0.03)A	2.27 (0.16)A	1.11 (0.01)A	1.75 (0.12)A
Fine root length density (m m^−3^)									
Lodgepole pine	Low	173 (23)B	215 (17)A	201 (39)A	176 (16)A	187 (18)B	233 (13)A	213 (41)A	190 (19)A
	Medium	392 (70)AB	472 (85)A	314 (87)A	183 (8)A	416 (71)AB	480 (85)A	330 (67)A	193 (9)A
	High	557 (23)A	607 (23)A	266 (105)A	170 (7)A	585 (29)A	633 (33)A	280 (103)A	188 (6)A
White spruce	Low	313 (51)A	334 (55)A	161 (23)A	179 (24)A	323 (51)A	390 (71)A	183 (20)A	189 (24)A
	Medium	393 (48)A	431 (41)A	183 (16)A	133 (17)A	401 (54)A	441 (39)A	191 (16)A	143 (16)A
	High	356 (19)A	405 (23)A	156 (5)A	150 (13)A	364 (19)A	421 (23)A	172 (6)A	170 (14)A
Fine root biomass (kg ha^−1^)									
Lodgepole pine	Low	322 (124)A	265 (123)A	190 (81)A	139 (30)A	366 (134)A	304 (64)A	161 (42)A	168 (30)A
	Medium	221 (33)A	268 (49)A	103 (3)A	47 (3)A	256 (37)A	285 (129)A	123 (9)A	72 (12)A
	High	269 (77)A	294 (80)A	348 (126)A	101 (34)A	299 (78)A	321 (81)A	231 (23)A	122 (48)A
White spruce	Low	85 (13)A	184 (3)A	151 (80)A	81 (6)A	107 (8)A	193 (5)A	167 (4)A	105 (19)A
	Medium	146 (21)A	179 (3)A	132 (23)A	117 (27)A	183 (37)A	219 (11)A	187 (8)A	97 (5)A
	High	253 (111)A	116 (1)B	118 (63)A	44 (13)A	333 (158)A	160 (12)A	116 (23)A	75 (6)A

a Means with different upper case letters indicate significant difference between stand productivity levels in each column.

Values are shown in brackets indicate standard error (*n = 6*).

Fine root length density was significantly influenced by productivity level and sampling time) in pine stands (Table 2, Table S1). It was higher in the high than in the low productivity sites in the first sampling in both 2011 and 2012. Fine root length density averaged over the 2 years varied by 2, −53, and −67% from low to high productivity level. It was not affected by productivity level in both 2011 (*P *=* *0.60) and 2012 (*P *=* *0.78) but was affected by time of sampling (*P *<* *0.05) in spruce stands in both 2011 and 2012. In both pine and spruce stands, mean fine root length density decreased by approximately 50% from June to September regardless of the productivity level except for the low productivity pine stand (Table 2).

The mean fine root biomass was not affected by productivity level for both species (Table 2, Table S1) in both 2011 and 2012, except in the July 2011 sampling in the white spruce stands. In general, in 2011, the lowest seasonal mean value (160 kg ha^−1^) was found in the medium productivity level and the highest seasonal value (253 kg ha^−1^) was found in the high productivity level in pine stands. In spruce stands, the lowest seasonal mean fine root biomass (125 kg ha^−1^) was found in the low productivity level and the highest seasonal value (144 kg ha^−1^) was found in the high productivity level. Mean fine root biomass was greater in 2012 than in 2011 in all three productivity levels of both species, except in the high productivity pine sites. From 2011 to 2012, mean fine root biomass varied by 6, 15, and −1.3% in low, medium and high productivity levels, respectively, in pine stands, and 14, 29, and 19%, respectively, in spruce stands. Mean fine root biomass in pine trees was significantly different among sampling months (Table 2, Table S1). The highest mean value was found in June (289 kg ha^−1^), and the lowest mean value was found in September (109 kg ha^−1^) across the productivity gradient and years in lodgepole pine stands. There were no significant differences among sampling times along the productivity gradient in spruce stands.

### Fine root decomposition

The fine root decomposition rate, quantified as the percent mass remaining, was not affected by productivity level but was affected by incubation time for both pine and spruce stands (Table 3). The average fine root mass remaining ranged from 36% to 87% in pine stands and from 41 to 86% in spruce stands across the incubation periods (data not shown). Percent total fine root mass loss was not altered by the productivity level in both pine (*P *=* *0.33) and spruce (*P *=* *0.30) stands. The k values were not different between species along the productivity gradient. Mean k values were in the order (as a pattern, *P* > 0.05) of low < medium < high in pine stands and low > medium > high in spruce stands, with the mean residence time followed an opposite trend to that of k values for both tree species (Table 4).

**Table 3:** Percent mass of fine roots of lodgepole pine and white spruce remaining after each incubation period along stand productivity gradients in oil sands reclamation

Stand productivity level	Lodgepole pine			White spruce		
	Oct. 2011	May. 2012	Jul. 2012	Oct. 2011	May. 2012	Jul. 2012
Low	95 (6.36) A^a^	76 (14.83) A	46 (4.24) A	86 (9.19) A	67 (9.90) A	39 (2.15) A
Medium	80 (0.71) A	42 (2.83) A	32 (1.41) A	94 (0.01) A	76 (2.12) A	51 (8.13) A
High	87 (2.12) A	46 (1.41) A	32 (3.54) A	79 (2.84) A	79 (6.36) A	32 (1.41) A
Repeated measures ANOVA						
	*F* value	*P* value		*F* value	*P* value	
Stand productivity	5.32	0.103		0.43	0.686	
Time of incubation	222.7	<0.001		71.46	<0.001	
Stand productivity * time of incubation	5.04	0.060		0.41	0.799	

a Means with same capital letters indicate nonsignificant differences between stand productivity in each column.

Values shown in brackets are standard errors of the mean (*n* = 8).

**Table 4:** Percent total fine root mass loss, decomposition rate (*k* value in year^−1^) and mean residence time in years in of lodgepole pine and white spruce stands along a stand productivity gradient in oil sands reclamation

Stand productivity	Lodgepole pine			White spruce		
	Fine root mass loss (%)	*k* value (year^−1^)	Mean residence time (year)	Fine root mass loss (%)	*k* value (year^−1^)	Mean residence time (year)
Low	54 (7.07) A^a^	0.190 (0.03) A	5.52 (0.87) A	61 (9.19) A	0.241 A	4.68 (1.10) A
Medium	68 (1.41) A	0.262 (0.01) A	3.84 (0.17) A	49 (8.12) A	0.175 A	6.12 (0.90) A
High	68 (2.83) A	0.265 (0.02) A	3.84 (0.34) A	68 (1.41) A	0.165 A	6.11 (0.42) A
One‐way ANOVA						
*F* value	1.63	1.73	1.63	2.29	1.64	2.25
*P* value	0.331	0.320	0.331	0.304	0.379	0.308

a Means with same capital letters indicate nonsignificant differences between stand productivity in each column.

Values shown in brackets are standard errors of the mean (*n* = 8).

### Total fine root production and turnover rates

**Figure 1:**
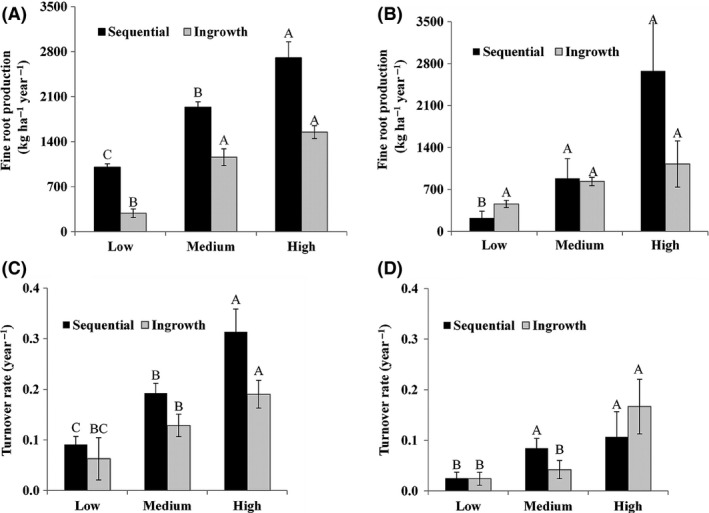
Fine root production in (A) lodgepole pine and (B) white spruce, and turnover rate in (C) lodgepole pine and (D) white spruce stands along a stand productivity gradient in oil sands reclamation measured by sequential and ingrowth core methods. Values are means ± SE (*n* = 24). Means across the productivity gradient within each method with the same uppercase letter are not significantly different at *P* < 0.05.

Total fine root production measured using the sequential core method was 1004 to 2704 and 225 to 2676 kg ha^−1^ year^−1^ in pine and spruce stands, respectively, along the productivity gradients (Fig. 1). It increased in pine stands with stand productivity (*P *<* *0.05) when measured by the sequential coring method, while was not affected by stand productivity in spruce stands (Fig. 1). Total fine root production was not different among the productivity levels in both pine (*P *=* *0.45) and spruce stands (*P *=* *0.37) when measured by the ingrowth core method.

Turnover rate measured with the sequential core method increased (*P *<* *0.05) with increasing stand productivity in pine stands (Fig. 1), but not in spruce stands (*P *=* *0.33). Turnover rate calculated using ingrowth cores did not change with stand productivity. Estimates of turnover rates in pine and spruce stands ranged from 0.04 to 0.32 and 0.02 to 0.16 year^−1^, respectively, across the measurement methods and productivity levels (Fig. 1). In general, the values were greater with the sequential coring than with the ingrowth core method.

### Soil, fine root, and tree performance parameters

For pine stands, significant relationships were observed between available nitrogen and fine root decomposition rate, but not between available nitrogen and other fine root properties such as fine root biomass, fine root surface area, fine root production, or turnover rates (Table 5). Fine root decomposition was significantly correlated with available nitrogen, fine root production, and turnover rates. Fine root production and turnover rates were correlated with each other (Table 5). In spruce stands, most variables of fine root properties were negatively correlated with soil compaction. Nitrogen availability was also negatively correlated with soil compaction. Total fine root biomass was positively correlated with soil pH (Table 5).

**Table 5:** Pearson correlation coefficient (*r*‐value) and significance+ among soil variables in lodgepole pine and white spruce stands in oil sands reclamation (*n* = 24)

Variable	Avail. N	Compaction	EC	pH	Water content	Soil temp	FRLD	FRP	RSA	FRB	TOR	MRT
Lodgepole pine												
Compaction	0.50*											
EC	−0.27	0.72*										
pH	−0.16	0.07	−0.45*									
Water content	0.55*	−0.52*	−0.04	0.44*								
Soil temp	−0.24	0.15	0.46*	−0.13	0.01							
FRLD	0.26	−0.50*	0.43*	0.27	0.38	0.13						
FRP	0.07	0.11	−0.52*	0.33	−0.28	−0.38	−0.1					
RSA	0.19	−0.04	−0.29	0.28	0.35*	−0.07	0.69*	−0.02				
FRB	0.34	0.19	‐0.37	0.50*	0.17	0.10	−0.1	0.85*	0.14			
TOR	−0.23	−0.32	−0.52*	−0.24	−0.52*	−0.28	−0.07	0.51*	0.13	−0.02		
FR loss.	−0.03	0.2	0.28	−0.02	0.12	0.45*	0.19	−0.19	−0.25	−0.04	−0.30	
MRT	−0.49	0.16	0.95*	−0.27	−0.03	0.49*	−0.37	−0.71*	−0.24	−0.35	−0.67*	
*k* value	0.62*	−0.23	−0.87*	0.10	0.03	−0.48*	0.33	0.71*	0.20	0.36	0.62*	−0.98*
White spruce												
Compaction	−0.66*											
EC	0.75*	−0.56*										
pH	−0.77*	0.36	−0.83*			0.55*						
Water content	−0.41*	−0.03	−0.44*	−0.45*		0.19						
Soil temp	−0.54	0.55*	−0.51*	0.54*								
FRLD	−0.08	−0.09	0.07	−0.05	0.14	0.33						
FRP	0.24	−0.05*	0.33	0.09	−0.02	−0.20	−0.07					
RSA	0.20	−0.32*	0.21	−0.17	0.63	0.08	0.82*	0.13				
FRB	0.04	−0.27	−0.15	0.52*	0.10	0.29	−0.17	0.82*	0.27			
TOR	−0.37*	−0.54*	0.49*	−0.13	−0.07	−0.24	−0.02	0.97*	0.14	0.66*		
FR loss.	−0.01	−0.14	−0.28	0.2	0.32	0.19	0.57*	−0.09	0.43*	0.12	−0.14	
MRT	0.81*	−0.42*	0.22	−0.42*	−0.26	−0.37	−0.18	0.14	0.11	0.05	0.12	
*k* value	−0.85*	0.49*	−0.31	0.38	0.22	0.34	0.19	−0.24	−0.12	−0.09	−0.17	−0.96*

^+^*Significant at the *P *<* *0.05 level.

Available N: available nitrogen, EC: electrical conductivity, FRLD: fine root length density, FRP: fine root production, RSA: root surface area, FRB: fine root biomass, TOR: turnover rates, FR loss: fine root loss, MRT: mean residence time, *k* value: decomposition rate.

### Fine root dynamics and their relationship with tree performance

Linear regression analysis indicated that total fine root length density, fine root production, and turnover rates linearly increased with tree height and DBH in pine stands. In spruce stands, there was no significant relationship between total fine root length density, fine root production, and turnover rates and any of the tree performance parameters (Table 6).

**Table 6:** Regression equations for the relationships between fine root properties (*y*) and tree performance (*x*) in oil sands reclamation (*n* = 6)

Fine root properties	Lodgepole pine		White spruce	
	Equation	*R* ^2^	Equation	*R* ^2^
Fine root length density and tree diameter at breast height	*y* = 121.7*x* − 467.7	0.88*	*y* = 9.0*x* + 297.3	0.12
Fine root length density and tree height	*y* = 231.4*x* − 637.7	0.97*	*y* = 11.2*x* + 298.2	0.13
Fine root production and tree height	*y* = 231.4*x* − 637.7	0.94*	*y* = 11.2*x* + 298.2	0.13
Fine root production and tree diameter at breast height	*y* = 121.7*x* − 467.7	0.76*	*y* = 0.11*x* + 0.50	0.04
Turnover rate and tree height	*y* = 231.4*x* − 637.7	0.98*	*y* = 0.01*x* + 0.06	0.04
Turnover rate and tree diameter at breast height	*y* = 121.7*x* − 467.7	0.89*	*y* = 0.01*x* + 0.01	0.15

^+^*Significant at *P *<* *0.05.

## Discussion

### Fine root production and turnover rates

Increased fine root production and turnover rate with increased stand productivity in the pine stands (Fig. 1) is consistent with Yuan and Chen (2013) and may be explained by changes in resource availability along the productivity gradient. According to the optimality theory (Espeleta and Donovan 2002), trees growing in a nutrient and water‐limited environment would be expected to maximize their nutrient uptake by increasing their fine root productivity and turnover rate. This has been demonstrated in previous studies that focused on water and nutrient limitations in reclaimed sites with tailings sand as a substrate (Naeth et al. 2011; Jung et al. 2014; Luna Wolter and Naeth 2014). Fine root growth is typically influenced by available nutrients in forest ecosystems (Ingestad and Agren 1991 **)** and given the significant response for fine root biomass and turnover rate across the productivity gradient that is likely the case in our study. However, fine root growth in pine stands in our study was not significantly correlated with nitrogen availability (Table 5), suggesting that there could be other factors such as water availability or salinity that affect fine root growth in PMM capping material over tailings sand (Duan et al. 2015). Duan et al. (2015) showed that water availability increased along the productivity gradient and subsequently improved the tree performance. However, it is difficult to conclude that the increase in fine root production and turnover rates along the productivity gradient in pine sites was associated with water availability as such relationships were not determined in this study.

The negative relationship (*P* < 0.05) between fine root productivity and turnover rate and EC in pine sites (Table 5) indicates that higher EC reduced fine root productivity and turnover rate in some of the pine sites. Fine root production in spruce sites might also be affected by the EC of overburden material as indicated in a previous study on the same study site showing EC was also a problem for the growth of spruce trees planted on an overburden substrate (Duan et al. 2015). However, in this study, fine root productivity in spruce sites was not related to EC (Table 5). In general, the increase in EC may impose two stresses on plant growth (Fageria et al. 2010): (1) the increased EC may increase the osmotic potential in the rhizosphere, which may cause water stress and subsequently affect plant growth (Munns and Tester 2008); and (2) toxic effects of high concentration of ions associated with high salinity may cause nutrient imbalance and affect plant growth (Grattan and Grieve 1992), including root growth. Lilles et al. (2012) suggested that reduction in root growth on sites with high salinity at depth may affect long‐term productivity of established forests. Khasa et al. (2002) found that lodgepole pine seedlings had lower survival at high salt concentrations. High EC levels can also reduce microbial activity and nitrogen mineralization (Pathak and Rao 1998), which can lead to reduced turnover rate. The lack of relationship between fine root productivity and turnover rate with available nitrogen may be linked to soil compaction in the overburden substrate into which spruce was planted. Soil compaction can reduce microbial activity and nitrogen mineralization (Tan et al. 2005), which in turn can restrict fine root growth and overall tree performance (Kozlowski 1999).

Fine root production and turnover rates in this study are on the lower end of the reported range (Yuan and Chen 2013). The sequential core method may underestimate fine root production rates relative to the ingrowth core method (Makkonen and Helmisaari 1999; Yuan and Chen 2013) and are affected by the length of sampling interval and loss of roots between two sampling periods (Finér and Laine 1998). The ingrowth core method takes a minimum of 12 months for roots to recolonize the cores in temperate forests (Hendricks et al. 2006). It may take longer to recolonize the cores in colder and more resource‐limited environments. More advanced methods such as the minirhizotron technique should be considered for root measurements in the future.

### Fine root decomposition

Our species‐specific differences in fine root decomposition could be due to differences in the degree of mycorrhizal colonization (Koide et al. 2011) as both pine and spruce are mycorrhizal species (Comeau and Kimmins 1989; Ostonen et al. 2011), chemical composition of roots (Chen et al. 2002) and differences in substrates used in oil sands reclamation. Pine is an early successional coniferous species and has a faster rate of colonization by mycorrhizae (Shishido et al. 1996). Mycorrhizal colonization can result in increased rate of root decomposition, as colonization of roots by mycorrhizae can stimulate nitrogen content of root tissues which may lead to frequent grazing by soil herbivores and result in faster fine root decomposition (Koide et al. 2011). Spruce is a late successional species and mycorrhizae have high persistence in its root system in oil sands reclamation (Onwuchekwa et al. 2014), which can lower fine root decomposition rates in spruce. The longer mean residence time in fine roots of spruce trees may be due to increased recalcitrance of carbon compounds (Lin et al. 2011), such as lignin, in root tissues. Spruce fine roots have lower water‐soluble extractives (3% on a dry weight basis), greater water‐soluble phenols (1%), and lignin (0.3%) concentrations than pine fine roots (Chen et al. 2002) which might also lower the decomposition rate of spruce fine roots. Slower decomposition of spruce fine roots may be due to stabilization of soil aggregates by overburden compaction (Hertel et al. 2009). Highly compacted overburden substrates lack aeration, results in reduced microbial activity and lower nutrient availability (Lazorko and Van Rees 2012) and can therefore affect fine root decomposition. These results corroborate with the negative relationship between soil compaction and decomposition rate of fine roots of spruce (Table 5). High temperatures in the tailings sand substrate could increase fine root decomposition rate in pine stands (Naeth et al. 2011) as indicated by the positive relationships between soil temperature and fine root mass loss in this study (Table 5). The change in soil temperature may affect microbial activity and the decomposition of fine roots (Priha et al. 2001). Soil textural differences may also alter soil thermal conductivity and heat capacity (Hillel 2005). Sandy materials have higher thermal conductivity and lower heat capacity than clayey materials (Hillel 2005). In general, soils with low heat capacity (tailings sand) could have a higher temperature relative to soils with a high heat capacity (overburden). These differences in temperature could affect fine root decomposition in tailings sand. In a concurrent study, House (2015) showed that tailings sand had higher soil temperature than overburden during 2011 and 2012. Therefore, the greater decomposition rate of pine than spruce roots was most likely related to the higher temperature in tailings sand.

In this study, we used the mesh bag technique which may underestimate the k value (Chen et al. 2002) and might have not differed with productivity levels within pine and spruce stands. Large soil organisms such as soil vertebrates and invertebrates cannot enter the mesh bag (Mcclaugherty et al. 1984) and the disturbance of rhizosphere microorganisms such as bacteria, fungi, and their associations such as mycorrhizal colonization during root sample preparation while putting them in the mesh bag may also delay fine root decomposition as they need time to re‐establish in the soil (Bradbury et al. 1998).

### Total fine root length density and mean root surface area

The different patterns of total fine root length density and mean root surface area for the two tree species indicated varied resource exploitation abilities over the productivity levels (Table 2). The greater increase in total fine root length density among pine than spruce stands at the same productivity level suggests that pine roots may proliferate more quickly in reclaimed soils than spruce roots, although results are likely associated with the physical environment of substrates (water limitation) used in oil sands reclamation (Jung et al. 2014). Tailings sand is typically characterized by a lack of micropores that prevent capillary rise of water to the PMM capping layer (Li et al. 2013). The tailings sand typically has a low water‐holding capacity (Khasa et al. 2005) and may cause reduction of fine root growth below the interface layer. The characteristically greater total fine root length density in pine stands may therefore confirm that lodgepole pine trees could grow in a water‐stressed environment, in a reclaimed ecosystem (Jagodziński and Kałucka 2010) such as in a tailings sand substrate. This could be associated with the mycorrhizal colonization capacity of pine trees (Kranabetter et al. 2006), which is known as an efficient way to exploit the nutrient and water resources (Jagodziński and Kałucka 2010). Mycorrhizal colonization alters the root length and root architecture (Eissenstat et al. 200) and increases belowground absorptive surface area for coping with water limitations (Metcalfe et al. 2008). These changes in root architecture and production would have ecological implications for plant establishment, survival and productivity in water‐limiting conditions (Auge 2001).

The differences in mean root surface area between species are likely associated with differences in soil compaction and salinity of the overburden material as shown with the negative relationships of root surface area and soil compaction in spruce. Highly compacted overburden materials do not facilitate root penetration and reduce the plant's capability to extract the large soil volume for resources such as was characteristic of the spruce stands below the capping material (Jung et al. 2014). Under these conditions, resource limitations may be compensated by increasing root surface area (Rewald et al. 2011). However, such a compensating mechanism would be an inefficient way to increase resource availability in the zone where resources are depleted (Jagodziński and Kałucka 2010).

### Total fine root biomass

Different patterns in total fine root biomass further show clear species‐specific differences in fine root dynamics between pine and spruce in each productivity level, except the medium productivity pine sites. These results are associated with the fine root biomass being negatively correlated with the fertility of soils in boreal forests (Helmisaari et al. 2002; Yuan and Chen 2010). Seasonal and interannual variations in fine root biomass in pine were likely associated with changes in soil water and temperature, consistent with the findings of Comeau and Kimmins (1989). In general, fine root biomass peaked in mid‐ to late summer and was lowest in the fall. This coincides with the reduced demand for resources in late summer and fall when leaf senescence begins (Brassard et al. 2009). The greater fine root biomass in June than in the later months suggests that there would be growth of fine roots in earlier months such as May, when the soil temperature starts to increase. In general, fine root growth of woody species exponentially increases with increasing soil temperature (Pregitzer et al. 2000; Steinaker et al. 2010). The positive relationship between temperature and fine root biomass (Table 5) in this study suggests that most root growth occurred in the growing season (June–September) in the oil sands, as soil temperature would be very low outside that period to allow substantial fine root production. The increase in soil pH appeared to be strongly related to the increase of fine root biomass in both species due to their positive relationship (Table 5). Yuan and Chen (2010) found fine root biomass increased with increased soil pH but decreased with soil nutrient availability. This might be due to inhibited microbial activity under low pH, while at higher pH, they can compete with fine roots for nitrogen (Brunner et al. 2002). Thus, fine roots may proliferate with increasing nitrogen availability and subsequently fine root biomass increased with pH. Hahn and Marschner (1998) found increased root growth with lime application was associated with improved nutrient supply and biological activity.

### Contrasting relationships between lodgepole pine and white spruce

The stronger relationships of fine root properties with stand productivity in pine than in spruce are likely associated with interspecies differences in rooting habits, growth potentials, tolerance to environmental stresses, and site‐specific differences in substrate materials (Jung et al. 2014; Duan et al. 2015). Lodgepole pine has a potential to thrive in extreme environmental conditions (Stuart et al. 1989) and site types (Pinno et al. 2012). Pine species are less sensitive to compaction (Bulmer and Simpson 2005) and more tolerant to nutrient deficiencies (Bothwell et al. 2001; Jung et al. 2014), salinity (Khasa et al. 2005), and water limitation. This is likely associated with the pine having a taproot system with vertical sinkers on well‐drained sites. Its roots can go deeper in the soil for extracting resources (Kranabetter et al. 2006) increasing root growth and tree productivity (Ostonen et al. 2011). Pine has a greater nutrient retranslocation efficiency and maintains adequate nitrogen status in resource‐limited soils during maturity stages (Miller 1995) than spruce; those might have contributed to increased tree growth in reclaimed oil sands soils and maintaining the relationship between aboveground and belowground pine tree performance in this study.

The lack of relationships between fine root properties and stand productivity in spruce is likely associated with its slow rate of growth (Khasa et al. 2002), shallow rooting habit (Burns and Honkala 1990), sensitivity to salinity (Renault et al. 1998), compaction (Bothwell et al. 2001), and nutrient deficiency (Duan et al. 2015). Staples and Van Rees (2001) indicated that spruce growth could be affected at EC levels > 0.5 dS m^−1^. The upward movement of salts through diffusion from capping materials such as a sodic overburden material, if the capping layer is thin (Kessler et al. 2010), would increase the EC level and affect root growth (Duan et al. 2015). This is consistent with previous findings that the reduction of height in spruce was linked to reduced spruce root growth in saline soils (Lilles et al. 2012). The morphology and structure of spruce roots varies with nitrogen availability (Krasowski and Owens 1999) and soil chemical properties (Bredemeir et al. 1995). Site difference is also important in affecting tree growth and relationships between fine root properties and tree growth. The reader is cautioned that the differences between pine and spruce sites were confounded between species and site differences. Therefore, when interpreting differences between the stand types, both species and site differences need to be considered.

## Conclusions

The contrasting relationships of fine root properties with stand productivity in pine and spruce stands demonstrate the need for species‐ and site‐specific management in oil sands reclamation. The selection of tree species and substrate material could affect the success of oil sands reclamation. This study showed that most fine root parameters systematically changed along the productivity gradient in pine stands but not in spruce stands. The species‐specific differences in fine root dynamics were likely due to differences in the properties of tailings sand and overburden substrates. Fine root dynamics were strongly linked to EC in lodgepole pine stands, but were more affected by soil compaction in white spruce stands. The negative relationships of EC and soil compaction with nitrogen availability indirectly influenced fine root dynamics of both stand types. Thus, the effects of EC and compaction on reduction in resource acquisition and their relationships to fine root dynamics in reclaimed oil sands sites should be evaluated in more sites in the future. Fine root dynamics of both species should be measured under similar conditions with either PMM over tailings sand or PMM over overburden, for selection of suitable substrate and tree species for sustainable ecosystem development after reclamation in the oil sands region.

## Conflict of Interest

None declared.

## Supporting information


**Table S1.** Repeated measures ANOVA of fine root paramters of lodgepole pine and white spruce stands along stand productivity gradients in oil sands reclamation.
