# Supplementary material for: Fine root dynamics in lodgepole pine and white spruce stands along productivity gradients in reclaimed oil sands sites
Source: Ecol Evol. 2015 Oct 2;5(20):4655–70. doi: 10.1002/ece3.1742 (PMC4670065; doi:10.1002/ece3.1742)
Supplement: Supplementary file 1 — Table S1. Repeated measures ANOVA of fine root paramters of lodgepole pine and white spruce stands along stand productivity gradients in oil sands reclamation. [file ECE3-5-4655-s001.docx]

**Fine root dynamics in lodgepole pine and white spruce stands along productivity gradients in reclaimed oil sands sites**

G.M. Jamro^1,2^, S.X. Chang^1^, M.A. Naeth^1^, M. Duan^1^ and J.D. House^1^

**Supporting information**

**Table S1.** Repeated measures ANOVA of fine root paramters of lodgepole pine and white spruce stands along stand productivity gradients in oil sands reclamation

| Root parameter | 2011 | | | | | | | |  | 2012 | | | | | | | |
| --- | --- | --- | --- | --- | --- | --- | --- | --- | --- | --- | --- | --- | --- | --- | --- | --- | --- |
|  | Stand productivity level | |  | Sampling month | |  | Stand productivity level × sampling month | |  | Stand productivity level | |  | Sampling month | |  | Stand productivity level × sampling month | |
|  | F value | *p* value |  | F value | *p* value |  | F value | *p* value |  | F value | *p* value |  | F value | *p* value |  | F value | *p* value |
| Lodgepole pine | | | | | | | | | | | | | | | | | |
| Fine root surface area | 1.77 | 0.220 |  | 2.35 | 0.128 |  | 4.91 | 0.072 |  | 1.15 | 0.351 |  | 1.92 | 0.185 |  | 1.82 | 0.185 |
| Fine root length density | 9.34 | 0.004 |  | 7.86 | 0.003 |  | 2.25 | 0.123 |  | 9.88 | 0.003 |  | 8.29 | 0.002 |  | 2.38 | 0.103 |
| Fine root biomass | 1.41 | 0.295 |  | 3.45 | 0.045 |  | 0.55 | 0.767 |  | 1.34 | 0.301 |  | 5.57 | 0.014 |  | 0.42 | 0.863 |
|  |  |  |  |  |  |  |  |  |  |  |  |  |  |  |  |  |  |
| White spruce | | | | | | | | | | | | | | | | | |
| Fine root surface area | 9.34 | 0.004 |  | 36.79 | 0.001 |  | 7.88 | 0.071 |  | 8.20 | 0.006 |  | 16.71 | 0.002 |  | 4.46 | 0.096 |
| Fine root length density | 0.52 | 0.600 |  | 27.29 | 0.001 |  | 0.54 | 0.776 |  | 0.25 | 0.784 |  | 24.41 | 0.001 |  | 0.38 | 0.874 |
| Fine root biomass | 0.02 | 0.989 |  | 1.03 | 0.426 |  | 0.84 | 0.563 |  | 0.23 | 0.800 |  | 1.63 | 0.246 |  | 1.03 | 0.453 |
